# Metabolic comorbidities and post-transplant outcomes in Metabolic dysfunction-Associated Steatotic Liver Disease (MASLD): a cohort study from a Brazilian tertiary center

**DOI:** 10.20945/2359-4292-2025-0274

**Published:** 2025-10-08

**Authors:** Felipe Ramos Caprini, Fernanda Fernandes de Souza, Ajith Kumar Sankarankutty, Roberta Chaves Araújo

**Affiliations:** 1 Clínica Médica, Hospital das Clínicas, Faculdade de Medicina de Ribeirão Preto, Universidade de São Paulo, Ribeirão Preto, SP, Brasil; 2 Divisão de Gastroenterologia, Departamento de Clínica Médica, Faculdade de Medicina de Ribeirão Preto, Universidade de São Paulo, Ribeirão Preto, SP, Brasil; 3 Divisão de Cirurgia Digestiva, Departamento de Cirurgia e Anatomia, Faculdade de Medicina de Ribeirão Preto, Universidade de São Paulo, Ribeirão Preto, SP, Brasil

**Keywords:** Cirrhosis, hepatic transplantation, metabolic syndrome, obesity, steatosis

## Abstract

**Objective:**

Metabolic dysfunction-associated steatotic liver disease (MASLD), formerly known as nonalcoholic fatty liver disease, affects approximately 38% of the global population. MASLD’s strong association with obesity and type 2 diabetes positions it as an increasingly prevalent indication for liver transplantation. Hence, this study sought to assess the prevalence of MASLD as an indication for liver transplantation, to characterize the clinical and epidemiological profile of the affected population, and to investigate the rates of post-transplant recurrence and de novo occurrence. We also compared survival outcomes between recipients with MASLD and other etiologies.

**Materials and methods:**

We conducted a retrospective analysis of 610 patients listed for liver transplantation at Hospital das Clínicas (University of São Paulo) between 2005 and 2015. Data regarding demographics, comorbidities, and post-transplant outcomes were collected from medical records. The statistical analysis encompassed both descriptive and inferential methods.

**Results:**

Out of 610 patients, 61 (10%) were diagnosed with MASLD-related cirrhosis, presenting a waitlist mortality rate of 42.6%. Among the 264 who received transplants, 36 (13.6%) had MASLD as the primary diagnosis. Post-transplantation, 58 recipients developed steatosis, with 82.8% of these cases being de novo allograft steatosis. Pre-transplant obesity and hypertension were identified as significant risk factors. Importantly, patients undergoing transplantation for MASLD showed lower survival rates compared to those with other etiologies.

**Conclusion:**

MASLD patients who undergo liver transplantation exhibit distinctive clinical outcomes and reduced survival rates. These findings underscore the critical need for targeted risk assessments and developing long-term strategies to enhance the prognosis for this increasingly common patient demographic.

## INTRODUCTION

Metabolic dysfunction-associated steatotic liver disease (MASLD), previously referred to as nonal-coholic fatty liver disease (NAFLD), affects approximately 38% of individuals worldwide ^(1)^. The prevalence of MASLD varies by region, reaching 30%-40% in some Western countries and 15%-30% in Asian countries ^(2)^. This condition is closely associated with obesity, type 2 diabetes mellitus (DM2), and metabolic syndrome, with an estimated 60%-70% of patients with DM2 and 90% of those with severe obesity affected by MASLD ^(3)^. The disease spectrum ranges from simple steatosis to more advanced forms, including steatohepatitis, fibrosis, and cirrhosis, with up to 20% of MASLD patients progressing to advanced fibrosis ^(4)^.

Liver transplantation is essential for managing end-stage liver disease, with primary indications having shifted in recent years. Chronic hepatitis C virus infection, previously a leading cause, has declined due to effective antiviral treatments ^(5)^. Conversely, MASLD has rapidly emerged as a significant indication ^(6)^, currently accounting for 18%-25% of liver transplants in Western countries, driven by the global obesity epidemic ^(7)^. MASLD, together with alcohol-related liver disease, has become the predominant cause of liver transplants in the United States, with a 170% increase in MASLD patients on the waiting list from 2004 to 2013 ^(8)^. These trends elucidate the evolving epidemiology of liver disease and its impact on transplantation practices.

MASLD, which is closely linked to insulin resistance, can be considered the hepatic manifestation of metabolic syndrome. Patients with MASLD on liver transplant waiting lists often present with comorbidities such as obesity, DM2, chronic kidney disease, and elevated cardiovascular risk. These conditions require thorough pre-transplant evaluation and management to assess the risk-benefit ratio of surgery, address postoperative complications, and reduce the risk of disease recurrence following transplantation ^(9)^. Despite the increasing prevalence of MASLD, few national studies have examined the frequency with which MASLD leads to liver transplant listing and the outcomes of these patients. Additionally, patients transplanted for other indications may develop *de novo* MASLD due to factors such as reversal of cirrhosis-associated catabolism and immunosuppressive therapy, which may exacerbate metabolic disorders and contribute to post-transplant metabolic syndrome.

Given the above, this study aimed to evaluate the prevalence of MASLD as an indication for liver transplantation in a tertiary care facility. It assessed the clinical-epidemiological profile and mortality of individuals with MASLD on the transplant waiting list. The study also investigated the recurrence of MASLD and the incidence of *de novo* MASLD in transplant recipients. Additionally, clinical characteristics, metabolic complications, and survival were compared between post-transplant patients with and without MASLD, and potential risk factors associated with the development of post-transplant MASLD were analyzed.

## MATERIALS AND METHODS

This study employed an observational retrospective design by reviewing the medical records of patients enrolled in and undergoing hepatic transplantation at the Liver Transplant Outpatient Clinic of *Hospital das Clínicas*, Ribeirão Preto Medical School, University of São Paulo. The study was approved by the institution’s ethics committee (protocol no. 47183221.4.0000.5440). We recruited patients aged ≥ 18 years who were listed for liver transplantation at hospital between 2005 and 2015. Indications for transplantation were defined according to national standards. Data were collected for the post-transplant period until December 2020 for those who underwent the procedure.

The recorded information included sex, age, diagnosis of primary liver disease, body mass index (BMI) calculated from corrected weight (after removal of ascites), model for end-stage liver disease (MELD) score, estimated creatinine clearance calculated using the Cockcroft & Gault formula ^(10)^, histories of DM2, arterial hypertension, dyslipidemia, cardiovascular disease, tobacco use (defined as any prior use before transplantation), alcohol consumption history, and mortality. Post-transplant clinical data encompassed anthropometric measurements such as weight and BMI at 1, 3, and 5 years after transplantation, immunosuppressive therapy, new diagnoses of DM2, hypertension, hyperlipidemia, cardiovascular events, development of allograft steatosis, and mortality. Post-transplant steatosis was assessed using either liver biopsy or imaging modalities, depending on clinical indication and availability. These methods were employed during follow-up to identify both recurrent and *de novo* steatosis in liver grafts; donor sex and age were also included. Multiple organ transplants were excluded from analysis because of the potentially different cardiovascular risks associated with kidney transplants.

Patients identified as having MASLD were originally diagnosed with NAFLD, the terminology used during the data collection period. Following a recent international consensus, MASLD has been proposed to replace NAFLD, offering a more accurate reflection of underlying metabolic dysfunction and associated risk factors. As more than 95%-98% of individuals previously classified as NAFLD also meet the criteria for MASLD, the retrospective use of updated terminology is considered methodologically appropriate and broadly accepted in the scientific literature, provided that the original diagnostic criteria are clearly acknowledged ^(11-13)^.

Mean, standard deviation, median, minimum, and maximum values were used as descriptive statistics for numerical variables. Categorical variables were described using absolute frequency (n) and relative frequency (%). For univariate inferential statistics comparing the MASLD and non-MASLD groups post-transplantation, we utilized the c^2^ or exact c^2^ test for categorical variables and estimated odds ratios (OR) with corresponding 95% confidence intervals (95% CI). For numerical variables, we applied the Kolmorov-Smirnov test for normality; based on the results, either Student’s t-test for independent samples (parametric) or the Mann-Whitney test (non-parametric) was applied. Binary logistic regression was used for multivariate analysis. The absence of propensity score matching was acknowledged as a limitation.

The Kaplan-Meier method was used for survival analysis. The log-rank test compared survival curves among risk factors. Cox proportional hazards regression was used for multivariate survival analysis. A *p*-value below 0.05 was considered statistically significant; the clinical and epidemiological relevance of the findings was interpreted based on hazard ratios and 95% confidence intervals. Statistical analyses were performed using the Statistical Package for the Social Sciences (v. 23.0) for Windows (IBM Corp., 2015).

## RESULTS

### General results

**Figure 1:**
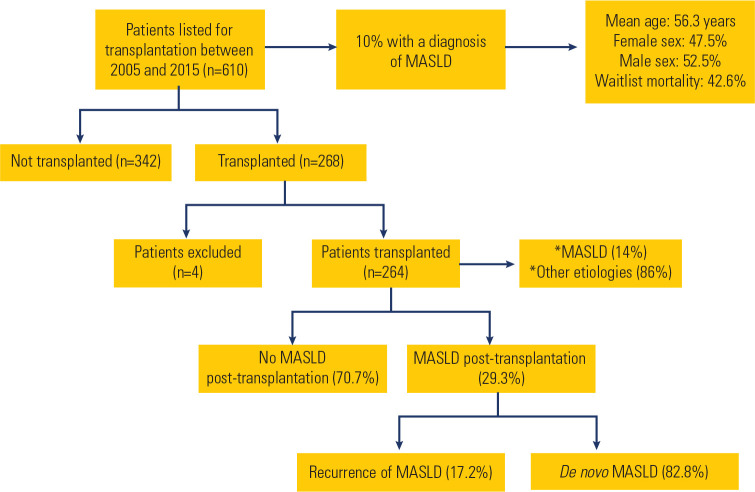
Characteristics of patients listed for liver transplantation (2005-2015) and summary of outcomes for transplanted individuals.

MASLD: Metabolic dysfunction-associated steatotic liver disease; *etiology of liver cirrhosis; n: number of patients included in the analysis.

We reviewed the medical records of 610 patients listed for liver transplantation between 2005 and 2015. Of these, 268 patients underwent transplantation, while 342 did not, either due to loss of clinical indication or death. Among all patients listed, 61 (10%) were diagnosed with liver cirrhosis secondary to MASLD. Of these, 29 (47.5%) were female and 32 (52.5%) were male, with a mean age of 56.3 years. The waitlist mortality rate for patients with MASLD-related liver cirrhosis was 42.6%. Four of the 268 transplanted patients were excluded from the study owing to insufficient medical records or lack of post-transplant follow-up information. The characteristics of the listed patients and the progression of transplanted patients to a diagnosis of hepatic steatosis are summarized in *Figure 1*.

### Clinical and demographic data of transplanted patients

The clinical and demographic characteristics of the 264 transplanted patients are summarized in *Table 1*. Approximately 76% of these patients were male, with a mean age at the time of transplantation of 52.4 ± 10.5 years and a BMI of 27 ± 5 kg/m^2^. The mean MELD-Na score was 19.4 ± 6.8.

**Table 1:** Clinical and demographic characteristics of the liver transplant recipients

*Variables*	*n*	*% (Mean - SD)*
Age (years)	264	53.7 ± 10.5
BMI (kg/m^2)^	263	27.0 ± 5.0
Male	264	76.5
White (race)	264	91.7
MELD score	264	17.7 ± 6.7
MELD-Na score	264	19.4 ± 6.8
Hypertension	264	28.8
Diabetes mellitus	264	23.5
Dyslipidemia	264	5.3
Chronic kidney disease	264	9.1
Cardiovascular disease	264	3.0
Obesity	264	26.5
Former alcohol use	258	71.3
Smoking	259	51.0

n: Number of patients included in the analysis; SD: standard deviation; BMI: body mass index.

**Figure 2:**
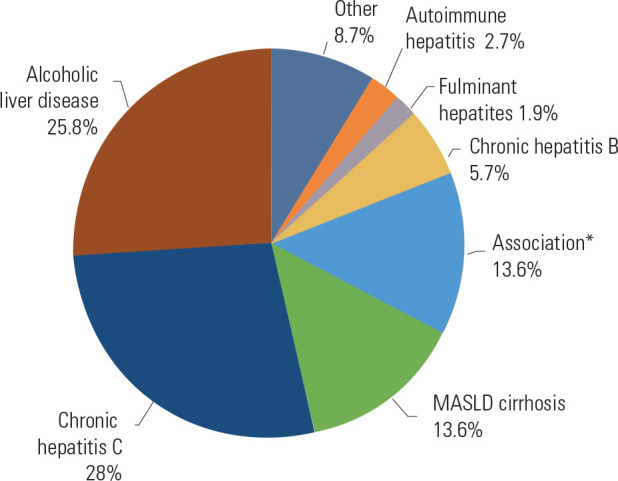
Primary diagnoses of patients undergoing liver transplantation from 2005 to 2015.

Among the 264 transplant recipients, chronic hepatitis C (28%) and alcoholic liver disease (25.8%) were the most prevalent indications. Thirty-six patients (13.6%) had liver cirrhosis due to MASLD. *Figure 2* illustrates the primary diagnoses among transplant recipients.

MASLD: Metabolic dysfunction-associated steatotic liver disease; *cirrhosis secondary to the association of more than one etiology, excluding MASLD.

Male patients predominated in the non-MASLD group (2.6 times more likely; OR = 2.61, 95% CI = 1.26-5.42), who also demonstrated higher odds ratios for alcohol and tobacco use. The group transplanted for MASLD exhibited higher MELD values (*p* = 0.01), and comorbidities including hypertension, DM2, obesity, and dyslipidemia were more prevalent in this group (*Table 2*).

**Table 2:** Comparison of comorbidities between individuals transplanted for liver cirrhosis secondary to MASLD and those with other etiologies

*Variables*	*Liver cirrhosis secondary to MASLD (%)*	*Liver cirrhosis secondary to other etiologies (%)*	*OR*	*95% CI*	*p-value*
Hypertension	45.9	26	2.4	1.19-4.92	0.01
Diabetes mellitus	45.9	19.8	3.4	1.67-7.09	0.001
Dyslipidemia	16.2	3.5	5.3	1.72-16.4	0.007
Obesity	45.9	23.3	2.8	1.36-5.7	0.004
Former alcohol use	47.2	75.2	3.4	1.65-6.9	0.001
Smoking	30.6	54.3	2.7	1.26-5.7	0.008

MASLD: Metabolic dysfunction-associated steatotic liver disease; OR: odds ratio. CI: confidence interval.

### Clinical and demographic characteristics of liver donors

Of the 264 donors, 60.7% were male, with a mean age of 38 ± 15 years and a mean BMI of 25.1 kg/m^2^ (±4.2). Regarding comorbidities, 27.8% had hypertension, 10.6% were classified as obese, and 4.2% had diabetes mellitus. Information on the presence and degree of steatosis was available for 156 donors; 11 (7%) had no steatosis. Among those with steatosis, the majority (78%) exhibited mild steatosis (in less than 30% of the liver parenchyma).

### Allograft steatosis and new comorbidities

Fifty-eight recipients exhibited steatosis identified via imaging or biopsy at least two months after liver transplantation. Of these, 48 (82.8%) developed *de novo* allograft steatosis, while 10 (17.2%) presented with recurrent MASLD. Univariate and multivariate analyses were performed to evaluate risk factors for allograft steatosis (*Table 3*). Among the metabolic risk factors analyzed (obesity, hypertension, dyslipidemia, and diabetes), obesity and hypertension were independently associated with allograft steatosis. In multivariate analysis, both conditions conferred nearly a twofold increased risk, highlighting their significant contribution to post-transplant metabolic complications.

**Table 3:** Risk factors for the development of steatosis in the allograft after liver transplantation

*Variables*	*Univariate analysis*			*Multivariate analysis*		
	*OR*	*95% CI*	*p-value*	*OR*	*95% CI*	*p-value*
Obesity	2.22	1.15-4.28	0.02	1.85	0.93-3.68	0.07
Hypertension	2.27	1.19-4.33	0.01	1.94	0.99-3.80	0.05

OR: odds ratio; CI: confidence interval.

Following transplantation, 22.5% of patients developed hypertension, while 26.5% developed DM2 as a new comorbidity. The prevalence of obesity post-transplant was 13.5% in one year, 22.5% in three years, and 21.4% in five years.

### Comparison between patients with recurrent and ***de novo*** MASLD

Patients who were obese three to five years post-transplant demonstrated higher rates of recurrent MASLD (OR = 7.75 and 10.8, 95% CI = 1.05-55.55 and 1.00-125.00, *p* = 0.06 and 0.05, respectively). The proportion of overweight patients was significantly higher among those who developed recurrent MASLD compared to those with *de novo* MASLD (75.0% vs 35.9%). Sex, age, hypertension, and DM2 were not determinants of progression to recurrent or *de novo* MASLD. No significant differences were observed in the type of immunosuppression used in the post-transplant period.

### Survival rate analysis

**Figure 3:**
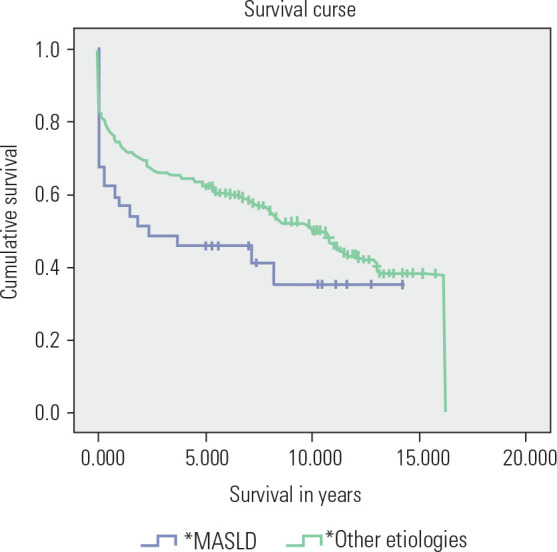
Comparative survival curve of individuals who underwent transplantation for cirrhosis due to MASLD and other etiologies.

The 1-, 3-, and 5-year survival rates of patients who received transplants between 2005 and 2015 were analyzed and compared by etiology. Of the 264 patients reviewed, 180 (68.2%) survived after one year, 159 (60.2%) after three years, and 152 (57.6%) after five years. The average survival was 8.69 years (95% CI = 7.79-9.58). Patients transplanted for MASLD demonstrated significantly different survival rates compared to those who received transplants for other etiologies (*Figure 3*).

MASLD: metabolic dysfunction-associated steatotic liver disease; *etiology of liver cirrhosis.

## DISCUSSION

MASLD has emerged as the most prevalent chronic liver disease globally over the past three decades. The number of patients on waiting lists and the number of liver transplants for MASLD have increased threefold in recent decades ^(14-16)^. In our institution, approximately 10% of patients listed for liver transplantation between 2005 and 2015 had a diagnosis of MASLD. The mortality rate among this group while on the waiting list was 42.6%. According to databases in Europe (European Liver Transplant Registry) and the United States (United Network for Organ Sharing), liver cirrhosis secondary to this condition accounted for 21.5% of transplant indications in North America in 2018, and 1.2% and 8.4% of cases in Europe in 2002 and 2016, respectively ^(9,17,18)^. Data on mortality rates for these patients while on waiting lists remain limited. The availability of donated organs and the patient’s clinical condition are the primary factors influencing the risk of death while awaiting transplantation. Additionally, patients with MASLD have a higher cardiovascular risk compared to those with other etiologies.

Liver cirrhosis secondary to MASLD was the primary diagnosis in 14% of transplant patients. Most transplant recipients had liver cirrhosis of other etiologies, with hepatitis C (28%) and alcoholic liver disease (25.8) being the most prevalent causes. This scenario can be attributed partly to the reduced efficacy of available hepatitis C treatment during the analyzed period, leading to a higher prevalence of this disease, as well as to limited awareness of MASLD, which affected the number of diagnoses. According to Narayanan and cols. ^(19)^, the predominant primary diagnoses among 588 patients who underwent transplantation between 1999 and 2006 were alcoholic liver disease (27%) and hepatitis C (23%), with MASLD present in 9% of cases.

One of the challenges in liver transplantation is donor steatosis, as a significant increase in this condition in the general population directly influences the growing prevalence of deceased and living donors with metabolic liver disease, which can exacerbate the overall shortage or quality of organs available for transplantation. In our study, donors had a mean BMI of 25 kg/m^2^, and up to 30% of organs exhibited mild steatosis. Spitzer and cols. ^(20)^ demonstrated that macrovesicular steatosis is an independent risk factor for graft survival; however, consensus is lacking regarding the percentage of steatosis at which primary graft function is definitively compromised. The most commonly used criterion considered low-risk for recipients is the presence of up to 30% macrovesicular steatosis in the organ ^(21-24)^.

In our study, 26.2% and 22.5% of patients developed DM2 and hypertension, respectively, during post-transplant follow-up, and 17.2% and 82.8% experienced recurrent and *de novo* MASLD, respectively. Evidence has indicated that rates of DM2 and hypertension post-transplant range from 10% to 64% and 50% to 100%, respectively ^(25,26)^, and that the incidence of recurrent MASLD ranges from 20% to 40%, influenced by the method used to detect hepatic steatosis ^(8)^. Advancements in surgical techniques and the development of immunosuppressive therapies have reduced mortality and improved post-procedure survival rates. Commonly prescribed immunosuppressive drugs are associated with the development of metabolic syndrome, making this condition a significant concern in the post-transplant period. As MASLD is a hepatic manifestation of metabolic syndrome, it is not unexpected that both recurrent and *de novo* metabolic liver disease can be observed during clinical follow-up of transplant recipients ^(8)^.

The risk factors identified for allograft steatosis, such as obesity and hypertension, were consistent with previous studies. According to Narayanan and cols. ^(19)^, in addition to obesity, female sex was also associated with post-transplant hepatic steatosis. Other authors have cited the development of post-transplant dyslipidemia and the history of alcohol abuse as risk factors ^(27)^. Notably, a primary diagnosis of MASLD prior to transplant was not an independent risk factor for allograft steatosis, possibly due to the smaller number of patients with this condition during follow-up.

The survival rate of patients who underwent transplantation for MASLD was significantly lower than that of individuals with liver cirrhosis due to other etiologies. Nagai and cols. ^(21)^ also reported reduced survival among recipients with MASLD compared to those transplanted for hepatitis C virus or alcoholic liver disease, identifying cardiovascular and cerebrovascular events as the major contributors to mortality. Although our study did not specifically analyze the cause of death, patients with MASLD-associated cirrhosis exhibited a higher prevalence of preexisting hypertension, diabetes, and obesity than those with other underlying liver diseases. These metabolic comorbidities, known to increase cardiovascular risk, likely contribute to the observed decrease in post-transplant survival in this population.

This study faced several limitations, including its retrospective nature, which relied on data from electronic medical records that may be incomplete or inconsistently reported. Moreover, the temporal context of the cohort, which corresponds to a period when hepatitis C was the predominant indication for liver transplantation, may constrain the generalizability of our findings to the current clinical environment. This is particularly relevant in the era of direct-acting antivirals, which have significantly decreased the prevalence of hepatitis C. Further studies incorporating more recent data are needed to better capture contemporary epidemiological trends and to allow a more accurate assessment of changes in the MASLD liver transplant population over time. Additionally, the relatively small number of cases with confirmed allograft steatosis, partially due to reliance on ultrasound detection, which typically identifies steatosis only when it affects at least 30% of the liver, represents another limitation. Despite these constraints, our results support the observation that post-transplant survival for patients with MASLD may differ from that of individuals with other etiologies. Future studies under the current MASLD nomenclature will be essential to validate these findings and provide a more comprehensive understanding of post-transplant outcomes for this population.

In conclusion, this study provides valuable insights into the clinical characteristics and post-transplant outcomes of patients with MASLD, which may exhibit distinct prognostic profiles compared with other liver disease etiologies. Despite its retrospective design and the diagnostic approaches employed at the time, the results indicate that patients with MASLD may face lower survival rates than those transplanted for other indications. Moreover, evidence suggests that individuals with MASLD displaying impaired pre-transplant functional status are at an increased risk of mortality and graft failure, emphasizing the clinical vulnerability of this subgroup ^(28)^. These findings highlight the importance of early identification and proactive management of metabolic comorbidities and functional assessment prior to transplantation. They may also contribute to the development of risk stratification strategies, guide individualized long-term care plans and support public health policies aimed at addressing the rising burden of metabolic liver disease in the transplant population.

## Data Availability

datasets related to this article will be available upon request to the corresponding author.
